# Clinical high-risk criteria of psychosis in 8- to 17-year-old community subjects and inpatients not suspected to develop psychosis: not pluripotential or transdiagnostic

**DOI:** 10.1192/j.eurpsy.2022.1752

**Published:** 2022-09-01

**Authors:** F. Schultze-Lutter, C. Michel, M. Franscini, N. Traber-Walker, P. Walger, B. Schimmelmann

**Affiliations:** 1 Heinrich-Heine University Düsseldorf, Department Of Psychiatry And Psychotherapy, Düsseldorf, Germany; 2 University of Bern, University Hospital Of Child And Adolescent Psychiatry And Psychotherapy, Bern, Switzerland; 3 University of Zürich, Department Of Child And Adolescent Psychiatry And Psychotherapy, Zürich, Switzerland

**Keywords:** pluripotent risk factor, transdiagnostic risk indicator, children and adolescents, clinical high risk

## Abstract

**Introduction:**

Based on high rates of non-converters to psychosis, especially in children and adolescents, it was suggested that CHR criteria were (1) pluripotential, (2) a transdiagnostic risk factor, or (3) simply a severity marker of mental disorders rather than specifically psychosis-predictive. If any of these three alternative explanatory models were true, their prevalence should differ between persons with and without mental disorders, and their severity should be associated with functional impairment as a measure of severity.

**Objectives:**

To compare the prevalence and severity of CHR criteria/symptoms in children and adolescents of the community and inpatients.

**Methods:**

We compared CHR criteria/symptoms in 8-17-year-olds of the community and of inpatients not clinically suspected to develop psychosis.

**Results:**

The 7.3%-prevalence rate of CHR criteria in community subjects did not differ significantly from the 9.5%-rate in inpatients. Frequency/severity of CHR criteria never differed between the community and the four inpatient groups, while the frequency and severity of CHR symptoms differed only minimally. Group differences were found in only four CHR symptoms: *suspiciousness/persecutory ideas* of the SIPS, and *thought pressure*, *derealization* and *visual perception disturbances* of the SPI-CY. These were consistent with a transdiagnostic risk factor or dimension, i.e., displayed higher frequency and severity in inpatients. Low functioning, however, was at most weakly related to the severity of CHR criteria/symptoms, with the highest, yet still weak correlation yielded for *suspiciousness/persecutory ideas.*

**Conclusions:**

The lack of systematic differences between inpatients and community subjects does not support suggestions that CHR criteria/symptoms are pluripotential or transdiagnostic syndromes, or merely markers of symptom severity

**Disclosure:**

No significant relationships.

